# The intricate mechanism of PLS3 in bone homeostasis and disease

**DOI:** 10.3389/fendo.2023.1168306

**Published:** 2023-07-07

**Authors:** Wenchao Zhong, Janak L. Pathak, Yueting Liang, Lidiia Zhytnik, Gerard Pals, Elisabeth M. W. Eekhoff, Nathalie Bravenboer, Dimitra Micha

**Affiliations:** ^1^ Department of Human Genetics, Amsterdam UMC Location Vrije Universiteit Amsterdam, Amsterdam, Netherlands; ^2^ Department of Clinical Chemistry, Amsterdam UMC Location Vrije Universiteit Amsterdam, Amsterdam, Netherlands; ^3^ Amsterdam Movement Sciences, Tissue Function And Regeneration, Amsterdam, Netherlands; ^4^ Department of Temporomandibular Joint, Guangdong Engineering Research Center of Oral Restoration and Reconstruction, Guangzhou Key Laboratory of Basic and Applied Research of Oral Regenerative Medicine, Affiliated Stomatology Hospital of Guangzhou Medical University, Guangzhou, Guangdong, China; ^5^ Department of Radiation Oncology, Peking University Cancer Hospital & Institute, Beijing, China; ^6^ The Second Clinical College, Guangzhou Medical University, Guangzhou, China; ^7^ Department of Traumatology and Orthopaedics, Institute of Clinical Medicine, The University of Tartu, Tartu, Estonia; ^8^ Department Internal Medicine Section Endocrinology and Metabolism, Amsterdam UMC Location Vrije Universiteit Amsterdam, Rare Bone Disease Center, AMS, Amsterdam, Netherlands; ^9^ Amsterdam Reproduction and Development Research Institute, Amsterdam, Netherlands

**Keywords:** PLS3, bone cells, mechanotransduction, calcium regulation, osteogenesis, bone diseases

## Abstract

Since our discovery in 2013 that genetic defects in *PLS3* lead to bone fragility, the mechanistic details of this process have remained obscure. It has been established that *PLS3* variants cause syndromic and nonsyndromic osteoporosis as well as osteoarthritis. PLS3 codes for an actin-bundling protein with a broad pattern of expression. As such, it is puzzling how PLS3 specifically leads to bone-related disease presentation. Our review aims to summarize the current state of knowledge regarding the function of PLS3 in the predominant cell types in the bone tissue, the osteocytes, osteoblasts and osteoclasts. This is related to the role of PLS3 in regulating mechanotransduction, calcium regulation, vesicle trafficking, cell differentiation and mineralization as part of the complex bone pathology presented by PLS3 defects. Considering the consequences of PLS3 defects on multiple aspects of bone tissue metabolism, our review motivates the study of its mechanism in bone diseases which can potentially help in the design of suitable therapy.

## 1 Introduction

PLS3 (plastin 3) is a cytoskeletal actin-binding protein encoded by the *PLS3* gene located on the X chromosome (1), whose main function is to participate in the bundling of F-actin to form parallel aligned F-actin bundles that mediate cell migration (2), endocytosis (2, 3), DNA repair (4) and membrane trafficking (5). PLS3 is commonly expressed in solid tissues; as such, it is also found in cells that directly influence bone development including osteoblasts, osteocytes, osteoclasts, as well as immune and angiogenic cells (1).

PLS3 is essential for the dynamic regulation of the actin cytoskeleton in the skeletal system which can adapt to gravity or mechanical stress changes through mechanotransduction. Mechanotransduction is the translation of mechanical signals into cellular responses, and its dysregulation is closely associated with bone-related diseases. Abnormalities in PLS3 have been suggested to be associated with nonsyndromic osteoporosis (6), osteogenesis imperfecta(OI) (7) and osteoarthritis (OA) (8–10). More specifically, alteration of PLS3 expression or function due to *PLS3* variants have been shown to cause osteoporosis and OI (7, 8). In contrast to osteoporosis and OI, studies have found increased levels of PLS3 in chondrocytes of patients with OA (8–10). Overall, a decrease or increase in PLS3 expression can lead to an imbalance in bone homeostasis and thus to the development of bone disease.

This review aims to specifically shed light on the bone-regulatory role of PLS3 by providing an overview of its function in the key player cells of the bone tissue. The role and function of PLS3 in these cells are examined in relation to mechanosensitivity, calcium regulation, bone mineralization, osteoclastogenesis, vesicle trafficking and immunity. We also aim to motivate the exploration of the unique pathological mechanism of PLS3, which can be expected to generate new insights in the molecular events leading to bone disease.

## 2 PLS3 regulates cell behavior by actin dynamics

**Figure 1:**
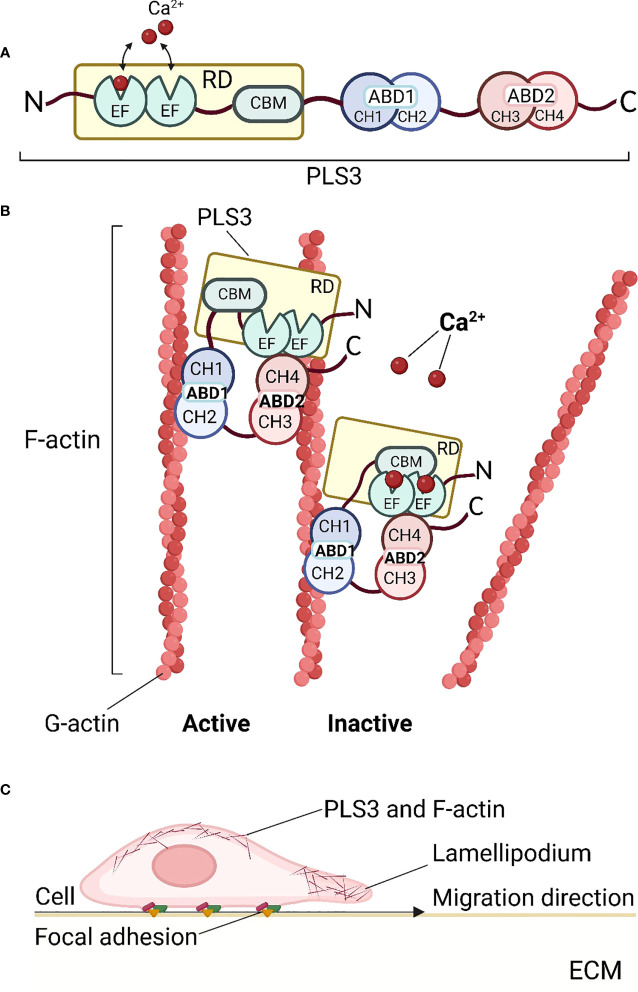
Structure and function of PLS3. **(A)** Schematic diagram of the PLS3 protein domain structure. The N-terminus of PLS3 is linked with the Ca^2+^-binding regulatory domain (RD), consisting of two EF-hand motifs and the calmodulin-binding motif (CBM). RD is followed by two actin-binding domains (ABD) - ABD1 and ABD2, both of which include two tandem calponin-homology (t-CH) domains, CH1, 2 and CH3,4 respectively. **(B)** Actin cytoskeleton remodeling by binding and bundling of PLS3. In the active state, when Ca^2+^ ions are absent, PLS3 binds two filamentous actin (F-actin) molecules, consisting of globular actin (G-actin) subunits. In the presence of Ca^2+^ ions PLS3 is inactive and the actin filament is released. **(C)** PLS3 and cell motility. As a component of the cytoskeleton involved in F-actin dynamics, PLS3 participates in cell migration through the extracellular matrix (ECM), focal adhesions, and actin cytoskeleton shaping near cell membranes. ABD1, 2, actin-binding domain 1, 2; CH1, 2, 3, 4, calponin-homology domain 1, 2, 3, 4; CMB, calmodulin-binding motif; ECM, extracellular matrix; EF, EF-hands domain; F-actin, filamentous actin; G-actin, globular actin; RD, regulatory domain. Figures were created with BioRender.com.

Plastin is an ancient and evolutionarily highly conserved family of actin-binding proteins that are expressed in a tissue-specific manner (1, 11). Three plastin isoforms are expressed in vertebrates: PLS1 (I-plastin) is expressed in absorptive intestinal and kidney cells (12), PLS2 (LCP, LCP1, or L-plastin) is predominantly found in hematopoietic and cancer cells (1, 13, 14), while PLS3 (T-plastin) is ubiquitously expressed in all solid tissues (1, 15). Interestingly, about 5% of the general population shows increased PLS3 expression in blood (16). All three plastin isoforms have modular structural domains, encompassing the N-terminal Ca^2+^-binding regulatory domain (RD) and a core consisting of two actin-binding domains (ABD1 and ABD2) (17) (**Figure 1A**). The RD contains two EF-hands and a calmodulin-binding motif (CBM), whereas each ABD is assembled from two tandem calponin-homology (t-CH) domains (17). The ABD cores form a compact and rather globular structure in an antiparallel arrangement induced by the contact between the CH1 and CH4 domains at the N- and C-terminal ends (11, 18). This conformational structure seems to be highly dynamic and dependent on the presence of Ca^2+^ (19). The conformational plasticity of CH2, within the structurally polymorphic ABD1, influences the diverse functions of different actin assemblies (20) (**Figure 1B**). Each ABD binds an individual filamentous actin (F-actin) molecule (21). F-actin is a polymer composed of monomeric globular subunits (G-actin) which is the major component of the cytoskeleton. Plastin is involved in the formation of the actin cytoskeleton through the binding and bundling of F-actin, which determines cell structure and behavior. However, the exact mechanism by which ABD1 and ABD2 bind to actin is unknown. It is currently thought that ABD1 is involved in F-actin binding while ABD2 is involved in bundling (22). *In vitro*, ABD1 alone is sufficient to positively affect Arp2/3-based actin dynamics (23). This can be potentially explained by the competitive replacement of tropomyosin enabling a higher filament recycling rate by cofilin (24), or the stabilization of filaments by competition with cofilin (23). Moreover, although actin-bundling the structural domains of both ABD1 and ABD2 are required for actin binding, defects in ABD1 alone can still lead to osteoporosis (17). To date, it is unclear whether plastin associates with the leading-edge of the actin cytoskeleton through the F-actin binding mode of ABD1 or the bundled mode of both ABD1 and ABD2 (17).

The cytoskeletal remodeling of actin plays a central role in regulating multiple components of cell behavior, including its morphology and motility. To control these functions, actin filament networks must be assembled, maintained, and disassembled at the correct time and place, through suitable filament organization and dynamics (25). The regulation of the actin networks has been largely attributed to the coordinated action of actin assembly factors by signaling cascades (25). PLS3 is involved in all cell processes dependent on F-actin dynamics such as cell motility (24, 26), focal adhesion (17), cell division (27), endocytosis (3), vesicle trafficking (3, 5), and motor axon outgrowth (28), in which it appears to be particularly important for shaping the actin cytoskeleton near the cell membrane (29). PLS3 strengthens and stabilizes cell membrane protrusions and promotes cell migration to enable bridging gaps in the extracellular matrix (ECM) (30). The formation of the PLS3/plectin/cofilin complex promotes cell migration and tube formation but weakens the adheren of junction formation in Ang II–treated endothelial cells (31). PLS3 is found to be associated with lamellipodia (17, 30) and focal adhesions in fibroblasts and osteoblasts (17). In the skin, PLS3 influences basement membrane assembly (32). The plastin ortholog in yeast (fimbrin) is localized almost exclusively in endocytic actin patches and is essential for endocytosis (24). Improved endocytosis has also been proposed to account for the role of PLS3 as a protective modifier of spinal muscular atrophy (28, 33–35). Furthermore, PLS3 mediates membrane trafficking in hypoxia (5)(**Figure 1C**). All the cellular functions listed above are the result of interactions between PLS3 and the actin cytoskeleton. Therefore, the involvement of PLS3 in the binding and bundling of F-actin is an essential part of cell structure and function.

## 3 PLS3 function in cells involved in bone metabolism

Studies on PLS3 mutation-induced osteoporosis and related animal models emphasize the importance of PLS3 in normal skeletal development and the maintenance of healthy bone homeostasis. But the specific mechanisms by which PLS3 affects osteogenesis and bone disease development remain unknown. Recently, the bone-regulatory role of PLS3 was demonstrated in some animal models. Knockout of *pls3* in zebrafish led to severe craniofacial abnormalities and muscle damage, as well as to malformed body axis and tail, which was reversed by restoring *PLS3* expression (7). *Pls3* knockout mouse models display impaired cortical thickness with decreased osteoblast mineralization capacity and defects in the development of the epidermal basal membrane (36). This contrasts with the bone thickening observed in *PLS3*-overexpressing mice which confirmed the key role of PLS3 in bone homeostasis, particularly in relation to osteoclast function (37). *PLS3* knockout rat model demonstrated an osteoporotic phenotypes with thinner cortices, significant low yield load, maximum load, and breaking load of femora (38). PLS3 protein was also found differentially expressed in dental pulp stem cells from deciduous teeth (SHED) during osteoblast development (39). Furthermore, based on the high expression of the PLS3 homolog fimbrin in chicken osteocyte dendrites (40, 41) and the significance of osteocyte dendrites for mechanosensing (40), it is tempting to hypothesize that *PLS3* variants cause loss of osteocyte mechanosensitivity, leading to osteoporosis.

PLS3 is known to be expressed in the three main bone cell types responsible for bone homeostasis, the osteocytes, osteoblasts and osteoclasts. However, PLS3 is the main plastin isoform expressed in osteoblasts and osteocytes (40, 42) whereas PLS2 is the main plastin isoform in osteoclasts (43). There are currently three hypotheses postulated about the mechanism of osteoporosis in patients with *PLS3* variants (1): dysregulation of osteocyte mechanosensing leading to misbalance between bone resorption and formation, (2) osteoblast mineralization insufficiency (44) and (3) increased osteoclast bone resorption (37). Elucidating the function of PLS3 in these three cell types will undoubtedly help us obtain a better understanding of PLS3 regulation in bone metabolism (7).

### 3.1 PLS3 in osteocytes

#### 3.1.1 Mechanosensory function of osteocytes

A substantial amount of evidence suggests that bone formation and bone resorption are coordinated by osteocytes buried within the mineralized bone (45) where they exert their unique mechanosensory function (46). Osteocytes are terminally differentiated osteoblasts located in osteocyte lacunae; their cell body contains 50-60 dendritic processes which radiate into the canalicular space. In this position, they are well-connected with neighboring osteocytes, osteoblasts, osteoclasts, blood vessels, nerve cells, and bone marrow, forming a complex osteocyte lacuno-canalicular system (47). Mechanical loading of bone triggers interstitial fluid flow through the lacuno-canalicular system, which causes shear stress to the osteocyte dendrites ultimately generating cellular signals (48, 49).

PLS3 is particularly enriched in the dendrites, specifically at their branching points (40, 42). In the mechanosensing dendrites, PLS3 is a critical component of the actin cytoskeleton, which facilitates osteocyte response to mechanical stimuli and their translation to signaling cascades (50); chemical depolymerization of actin results in dendrite retraction and a decrease in overall cell body size which demonstrates the dependence of osteocytes to actin organization for the maintenance of cytoskeletal integrity (41). In response to mechanical stimuli, nitric oxide (NO), prostaglandins (PGs) and within milliseconds ATP are released by osteocytes, which affects many other cellular signaling pathways including interleukin-6 (IL-6), receptor activator of nuclear factor κB ligand/osteoprotegerin (RANKL/OPG), Wnt/β-catenin and calcium signaling pathways (51, 52).

**Figure 2:**
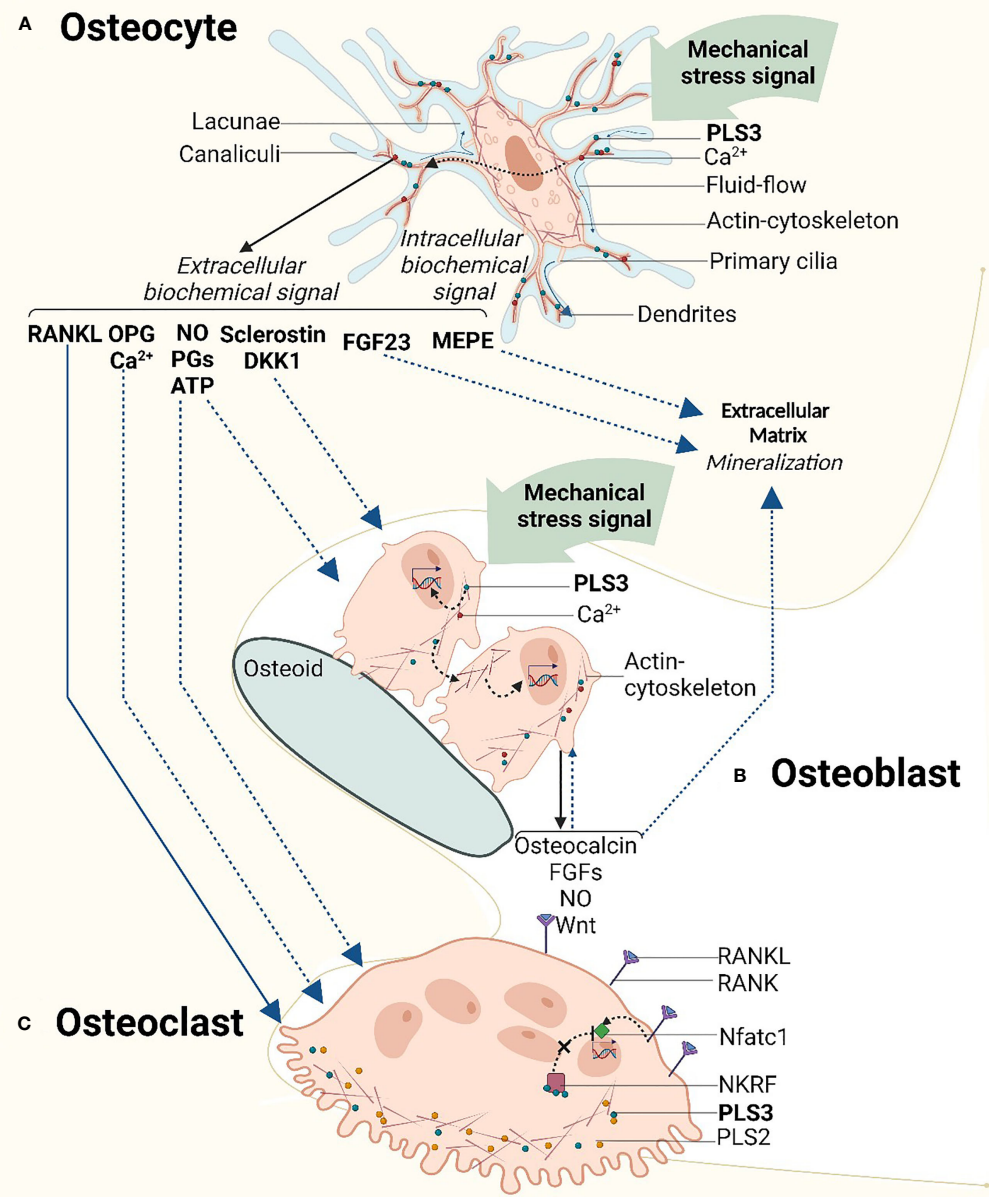
Schematic overview of the mechanosensory signaling cascade in relation to the cellular function of PLS3 in bone cells. **(A)** Mechanosensory function of PLS3 and calcium ion (Ca^2+^) oscillation in osteocytes. As a component of the mechanosensing actin cytoskeleton which is located in osteocyte dendrites, PLS3 reacts to mechanical stimuli activating a cascade of intracellular biochemical signals. Mechanical loading-induced Ca2+ oscillation triggers downstream signaling molecules of extracellular signaling pathways of bone metabolism. **(B)** Mechanical loading and PLS3 effects on osteoblasts and extracellular matrix mineralization. Mechanical stress in osteoblasts activates the production of signal molecules and growth factors similar to osteocytes, inducing the mineralization of the extracellular matrix. **(C)** TPLS3 in osteoclasts. PLS3 regulates osteoclast activity *via* the NFκB signaling pathway. DKK1, Dickkopf WNT Signaling Pathway Inhibitor 1; FGFs, fibroblast growth factors; FGF23 - fibroblast growth factor 23; MEPE, matrix extracellular phosphoglycoprotein; Nftac1, nuclear factor activated T cells c1; NKRF, NFκB-repressing factor; NO, nitric oxide; OPG, osteoprotegerin; PGs, prostaglandins; PLS3, t-plastin; RANK, receptor activator of nuclear factor κB; RANKL, receptor activator of nuclear factor κB ligand. Solid lines indicate pathways with solid evidence. Dashed lines indicate pathways with emerging evidence. Figures were created with BioRender.com.

Some studies mention that *PLS3* variants may potentially interfere with the mechanosensing function of osteocytes, which leads to bone homeostasis imbalance (53, 54). This is plausible considering that PLS3 regulates the dynamic organization of the actin cytoskeleton on which the osteocytes rely for their mechanosensory function. PLS3 defects may affect the mechanosensory process not only by the dendritic processes but also by the primary cilia on the osteocyte cell body (55). It can be also expected that focal adhesions participate in the PLS3-mediated cytoskeleton machinery for mechanosensing (56, 57). Focal adhesions are macromolecular complexes consisting of multiple actin-associated proteins, such as paxillin, vinculin, connexin-43, integrins, and talin that serve as physical linkages between the cell’s cytoskeleton and the ECM. Deletion of specific integrin subunits (β1 or β3) leads to attenuated mechanosensitivity in osteocytes *in vitro* but does not lead to a complete loss of mechanosensing *in vivo (* 56). The distribution of paxillin in the skull and fibula is reported to be different in osteocyte cell bodies *in situ* due to different external mechanical loading patterns; thus the mechanical loading pattern likely determines the orientation of the osteocyte actin cytoskeleton which may be disrupted in PLS3 defects (58) (**Figure 2A**).

#### 3.1.2 Calcium regulation in osteocytes

Ca^2+^ oscillation in osteocytes is a key regulator of their mechanotransduction function (59). Loading-induced Ca^2+^ oscillation in osteocytes triggers the release of downstream signaling molecules, e.g., NO (60), prostaglandin E2 (PGE2) (61), matrix extracellular phosphoglycoprotein (MEPE), insulin-like growth factor-1 (IGF-1) (62), and β-catenin (63), which modulate bone metabolism. Ca^2+^ levels in osteocytes and osteoblasts determine bone metabolism regulation and the rise of cytoplasmic Ca^2+^ is linked to bone development *via* calcineurin/NFAT57 and noncanonical Wnt53 pathways (42).

*PLS3* mutations were observed to lead to abnormal Ca^2+^ regulation in U2OS human osteosarcoma cells and OCY454 osteocytic cells (17). Cell behavior was investigated in five OI variants that are expected to produce full-length PLS3 protein; these included 2 variants in the loop between the two CH domains of ABD1, one variant in the second CH domain of ABD1 and 2 variants in the first CH domain of ABD2. Interestingly, one of the ABD1 loop variants led to Ca^2+^-hyposensitivity whereas the other variants showed Ca^2+^-hypersensitivity. The variant in the second ABD1 CH domain conferred Ca^2+^-hyposensitivity whereas of the other two variants in the ABD2 domain, one caused Ca^2+^-hyposensitivity and the other was the only one abolishing actin-bundling ability (18). This study also reported that in the Ca^2+^-hypersensitive cells, mutant PLS3 localized exclusively at the focal adhesions/stress fibers, which displayed reinforced morphology. However, in the Ca^2+^-hypersensitive cells, mutant PLS3 was restricted to lamellipodia, while chelation of Ca^2+^ caused its redistribution to focal adhesions (29). In striking contrast, wild-type PLS3 was distributed between the lamellipodia and focal adhesions, co-localizing with the F-actin-rich elements at the cell edge, focal adhesions, and stress fibers of osteoblasts and osteocytes. Although further conclusions cannot be made about the mechanism of the different ABDs in actin regulation, it can be deduced that part of the PLS3 OI mechanism entails the disruption of Ca^2+^ regulated plastin activity and localization across cytoskeletal structures (18) (**Figure 2A**).

Another study identified the binding of PLS3 to PLS2 which facilitates the Ca^2+^ release upon cell demand; a variant in the first EF-hand domain of PLS3 disrupts this interaction which weakens the regulation of intracellular Ca^2+^ in hFOB 1.19 cells (37), potentially contributing to osteoporosis. In conclusion, mechanical stimuli regulates the cytoplasmic Ca^2+^ rise in these cells which is essential for their translation to a cell response (64). *PLS3* variants may lead to the disruption of intracellular Ca^2+^ homeostasis which may have consequences not only directly on the function of PLS3 itself but also on other Ca^2+^-dependent signal transduction pathways.

#### 3.1.3 Signaling proteins produced by osteocytes

High serum concentrations of Dickkopf WNT Signaling Pathway Inhibitor 1 (DKK1) have been found in patients with *PLS3* variants (65), which is accompanied by hypomineralization of their bone matrix. Interestingly, no variations were observed in sclerostin and FGF23 in these subjects. Recently, a Finnish group found that lipocalin-2 is associated with FGF23 in 14 osteoporotic patients with *PLS3* pathogenic vairants. Although comparison was made for FGF23 levels between in *PLS3* patients and healthy subjects (66). DKK1, sclerostin, an inhibitor of the WNT signaling pathway, and FGF23 are primarily secreted by the osteocytes (67, 68). Drug-induced disruptions in the actin cytoskeleton and focal adhesion signaling have been reported to impact DKK1 mRNA levels in tumor cells (69). Thus, a possible explanation for the increased DKK1 production and secretion in PLS3 OI can be the cytoskeletal changes and osteocyte dysfunction induced by *PLS3* variants or other unknown pathways (65). On the contrary, serum microRNA profiles from 15 members of Finnish families with four different *PLS3* variants associated with osteoporosis, showed variation in the regulation of seven miRNAs; of these 2 upregulated miRNAs targeted DKK1 (70), and they promoted osteogenic differentiation by blocking DKK1 (71). These seven miRNAs were also observed to be enriched in the WNT pathway (70). It has been suggested that patients with *WNT1* and *PLS3* variants may share similar mechanisms in osteoporosis development (72). The specific mechanism of how *PLS3* variants interact with DKK1 and other components of WNT1 signaling to affect signal transduction pathways in osteocytes for the synthesis and secretion of related proteins needs to be further investigated.

Impaired osteocyte function leading to disruption of osteocyte-associated signaling protein production is known to lead to bone disease, including rare bone diseases (73, 74). Osteocyte-specific release of signaling molecules is disturbed during long-term unloading, such as when it occurs in astronauts during space travel and long-term bed rest (48). In particular, the production of sclerostin and the osteoclast stimulator RANKL by OCY454 osteocytes is upregulated in the absence of loading (75). Physiological loading has been shown to block osteocyte apoptosis (76), in contrast to reduced mechanical loading in a rat tail suspension model that increased osteocyte apoptosis (77). Data acquired from five *PLS3* variant carriers showed increased levels of apoptotic osteocytes together with abnormal gene expression of osteocyte-related genes including *FGF23*, *DMP1*, sclerostin and phosphorylated (phospho-) b-catenin (78) (**Figure 2A**). Thus, we can speculate that *PLS3* variants disrupt the mechanosensory function of osteocytes equivalently to unloading, by interfering with the release of relevant signaling proteins by osteocytes and shifting the bone homeostasis balance to catabolism that results in the poor bone mass in patients with PLS3 OI.

### 3.2 PLS3 in osteoblasts

A relation between osteoblast-induced bone mineralization and PLS3 has been postulated (44, 79). In healthy bone conditions, PLS3 abundance increases upon osteoblast differentiation (44, 53, 65, 80). However, in some patients with *PLS3* variants, matrix mineralization and osteoblast numbers are decreased and significant hypomineralization of the bone matrix is observed. Fahiminiya found that *in vitro* differentiation of cultured mouse cranial MC3T3- E1 osteoblasts correlated with increased expression of PLS3 (44), indirectly suggesting that PLS3 may be involved in bone mineralization. Regulation of intracellular vesicles in late osteoblasts transitioning to early osteocytes is thought to drive bone mineralization (81, 82). PLS3 is recognized as a regulator of vesicle trafficking (3, 5) and it is upregulated in matrix vesicles and microvilli of osteoblasts upon mineralization (79, 83). *Ex vivo* PLS3-deficient primary osteoblasts were found to display moderately impaired mineralization capacity (36). In these cells, the expression of *Sfrp4*, a gene associated with bone cortical formation or remodeling, was significantly reduced (36); interestingly, *SFRP4* variants lead to Pyle disease, a bone disorder characterized by thinning of the cortex (84). Here it is also important to state that no major abnormalities in Pls3-deficient osteoblasts were observed in terms of morphology, adhesion or migration assays (36). Other studies have shown that osteoblasts rely on high Ca^2+^ concentrations, which may in part be regulated by the interaction of the Ca^2+^-binding proteins PLS3 and PLS2; bound Ca^2+^ is released to increase the intracellular Ca^2+^ level when the extracellular Ca^2+^ level is low (85) (**Figure 2B**). Variants in the Ca^2+^ binding domains (EF-hands) of PLS3 weaken the interaction of PLS3 with PLS2 and thus might contribute to the osteoporotic phenotype. Future research can be anticipated to delineate the specific molecular mechanism of PLS3 affecting osteoblast-induced bone mineralization.

Osteoblasts can also respond to mechanical loading to stimulate bone modeling and remodeling (86). Pulsating fluid flow can trigger osteoblasts *in vivo* to produce a series of signaling molecules and growth factors, such as Wnt (87), NO (88), transforming growth factor β (89) and FGFs (90). Osteoblasts may stimulate bone formation either through the production of signaling molecules affecting neighboring cells or through mechanical stimulus-mediated osteogenic differentiation, for example by cytoskeletal alterations (91) (**Figure 2B**). In addition, it has been suggested that specific cortical bone phenotypic defects in PLS3-deficient mice may be due to the disruption of the specific cytoskeletal rearrangement, which interferes with the alignment of osteoblasts on the cortical bone surface (36), although the underlying molecular mechanism remains unclear.

As stated earlier, *PLS3* variants are known to disrupt the actin cytoskeletal integrity by impacting its ability to bind and bundle F-actin. This may affect the mechanical loading perception of osteoblasts which in turn can affect the production of osteocalcin (92) and FGFs (93), involved in calcium homeostasis and bone mineralization.

### 3.3 PLS3 in osteoclasts

Osteoclasts are known for their bone resorption function, and their hyperfunction or decline can lead to bone-related diseases by affecting directly or indirectly the behavior of other bone cells. Although the main plastin isoform in osteoclasts is PLS2, the precense of PLS3 in mice osteoclasts has been also proven (29). Recent work has proposed that PLS3 regulates osteoclast activity through the NFκB signaling pathway (37). NFκB signaling is a master regulatory pathway in osteoclastogenesis; it is initiated by the binding of NFκB ligand (RANK-L) to the RANK receptor on the surface of osteoclasts to activate the transcription of nuclear factor activated T cells c1 (Nfatc1), a key transcription factor regulating osteoclastogenesis (94). Nfatc1 expression is negatively regulated by the NFκB-repressing factor (NKRF). PLS3 inhibits osteoclastogenesis by binding to NKRF. PLS3 KO leads to insufficient inhibition of NKRF1 nuclear translocation, which results in increased osteoclast differentiation (37). Osteoclast resorption activity, migration, and adhesion are dependent on the formation of large actin filaments containing ring structures known as podosomes (95). These structures are impaired under the lack of PLS3 expression potentially due to F-actin depolymerization, leading to cytoskeletal instability (37). Compared with PLS3 KO, PLS3 overexpression may lead to increased transport of NKRF to the nucleus, thereby inhibiting Nfatc1 transcription and thus inhibiting osteoclastogenesis (37). Importantly, similarly to PLS3 KO, defects in podosome formation were also detected in PLS3 overexpressing mice, which may be caused by the reduced degradation of F-actin (37). This suggests that a certain level of PLS3 is critical for osteoclast structure and function. However, other studies have shown that PLS3 does not affect osteoclastogenesis (44). Osteoclast number was unchanged in the PLS3 KO mice (36). In the mean time, in transiliac bone biopsies from two patients with *PLS3* mutation-induced osteoporosis, no osteoblasts or osteoclasts were detected and the erosion surface was reduced (53).

Similarly to other bone cell types, Ca^2+^ is also a contributing factor in osteoclast function. As previously stated, a relatively high concentration of Ca^2+^ is essential for the differentiation of osteoblasts, while high extracellular Ca^2+^ inhibits osteoclast formation by acting on the calcium-sensing receptor of osteoclast precursor cells (96); this clearly suggests different expression profiles and roles for PLS3 in osteoblasts compared with osteoclasts (85) (**Figure 2C**).

## 4 PLS3-related bone disorders

Based on the above, it is clear that maintaining a precise balance of PLS3 in cells is essential for the health of the skeletal system (97). *PLS3* variants have been found to cause osteoporosis and OI (6, 7), while abnormally high levels of PLS3 have been associated with OA (8–10). Considering the wide expression pattern of PLS3 in the body, PLS3 also affects the presentation of other pathology types. PLS3 overexpression plays a protective role in several neurodegenerative diseases associated with decreased F-actin levels (16, 98–100), whereas S-nitrosylation of PLS3 is linked to thoracic aortic dissection (31). Moreover, PLS3 is involved in cancer development and it has been identified as an important predictor of the effectiveness of chemotherapeutic agents (101–103). However, this review aims to exclusively focus on PLS3-related bone diseases, including OI, osteoporosis, and OA.

### 4.1 OI and osteoporosis due to PLS3 variants

In 2013 we discovered variants in *PLS3* in five families as a cause of osteoporosis and fractures (7). Since then more families, with a total of 30 *PLS3* variants, have been discovered (https://www.LOVD.nl/PLS3), but the specific mechanism leading to disease development is limitedly deciphered. The severity of clinical manifestations in OI does not appear to be directly associated with specific *PLS3* variants, varying greatly from the absence of physical symptoms to severe early-onset osteoporosis and deformity (104). Although *PLS3* variants were initially described as a cause of monogenic nonsyndromic osteoporosis, recent reports have suggested that they can also cause OI (7). Syndromic extraskeletal OI manifestations have been reported in OI patients with PLS3 pathogenic variants, however, these reports are still scarce or describe subclinical manifestations, so there is some discrepancy as to whether PLS3 is a bone fide OI gene (105). Recently two OI patients with *PLS3* pathogenic variants were found in a comparative study of 140 Turkish OI families (106). Considering that *PLS3* is a relatively newly discovered gene, we believe that the full extent of its phenotypic spectrum may not be clear yet. Future studies are also expected to shed light on the underlying defect of syndromic OI characteristics, such as the blue sclera which is typically attributed to collagen defects in OI (107).

Regarding the nature of *PLS3* variants that have been reported in these patients, these include missense and nonsense variants, but also partial or total deletions (80, 108) and partial gene duplication (109). Reported pathogenic PLS3 variants are summarized in the LOVD OI variant database (https://www.LOVD.nl/PLS3) (110). Our study determined that *PLS3* variants are loss of function, causing either PLS3 deficiency or functional defects (7). Considering the ubiquitous expression of PLS3 in solid tissues, equally puzzling to the presence of the phenotype in the bone tissue is its absence in unaffected tissues. Tissue-specific dependency or functional redundancy in these healthy tissues may account for this. A remaining question is to which extent other plastins can compensate in the absence of functional PLS3. A study found that the abnormally high level of PLS3 Ca^2+^ sensitivity leading to osteoporosis is nearly identical to the physiological sensitivities of PLS2 and PLS1 (22). Another study on spinal muscular atrophy (SMA) showed that PLS2 was able to perform the same role as PLS3 in motor axons, thereby substituting for PLS3, albeit less efficiently, while PLS1 showed no ability to rescue these defects even though PLS1 could also bind and bundle actin (34).

Regarding the bone properties of patients with *PLS3* variants, bone biopsy studies have helped to provide some clues about the mechanism mediating the presentation of this type of osteoporosis. In a recent study of Kämpe et al., two children with disease-causing *PLS3* variants showed multiple peripheral and spinal fractures and low bone mineral density (BMD); iliac crest bone biopsies confirmed low-turnover osteoporosis in both patients (80). In another study one of the patients showed low bone turnover and low osteoid formation (105); in sharp contrast, children with OI type I tend to have elevated indices of bone formation and resorption (111) that were definitively not observed in the present case. However, the decreased trabecular bone volume and the abnormally high mineral content of the bone matrix are consistent with the characteristics of patients with PLS3-related OI (111). It is suggested that the increased matrix mineralization in this patient results primarily from a long history of very low bone turnover (105). Interestingly, the highly trabeculated cortical envelopes observed in this patient, indicated that bone modeling remains active, which may suggest that the ongoing cortical bone formation possibly represents a compensatory mechanism to counteract the lack of an adequate trabecular bone amount (105). It was recently reported that the PLS3-deficiency in mice predominately affects the early stage of cortical bone formation (36). Furthermore, a study demonstrated that children with *WNT1* or *PLS3* variants had heterogeneous bone matrix mineralization, consistent with bone modeling during growth. Bone matrix mineralization was homogenous in adults and increased throughout the age spectrum (112). We hypothesize that the unique function of PLS3 in different stages of skeletal development and the existence of compensatory mechanisms for the counteraction of the low bone quality caused by *PLS3* variants may lead to some heterogeneity in bone tissue biopsy analyses from these patients. Due to the wide variety of *PLS3* variants and the limitations posed by the early age of onset and invasiveness of biopsies, clarifying the genotype-phenotype correlation of bone material properties in OI patients is challenging. Overall, the very low and still undetermined prevalence of *PLS3* variants, their diversity, and the complexity of the genetic and clinical phenotypes add a degree of complexity in fully understanding the specific role of PLS3 in bone metabolism.

In addition to monogenic osteoporosis, PLS3 is also implicated in multifactorial age-related osteoporosis. Our study identified the rare *PLS3* variant rs140121121 which is associated with a risk of fracture among elderly heterozygous women; this was two times as high as that among noncarriers (7). Another study with a postmenopausal Chinese women cohort also demonstrated the association of *PLS3* variants with osteoporotic fractures supporting a role for PLS3 also in bone fragility with a polygenic etiology (113). Thus, delineating the role of PLS3 in monogenic osteoporosis might help toilluminate the path toward understanding the very prevalent age-related osteoporosis.

### 4.2 PLS3 in osteoarthritis

OA is a multifactorial disease characterized by predominantly articular cartilage damage and altered homeostasis within the extracellular matrix (114), an important component of articular cartilage function that is implicated in cartilage mechanotransduction. The molecular mechanisms involved in the development of OA remain obscure. It has been shown that osteoarthritic hypertrophic chondrocytes are primarily involved in the biological processes of cytoskeletal rearrangement, cell-cell and cell-ECM interactions during disease progression (115). Tsolis et al. found overexpression of PLS3 in chondrocytes in knee cartilage samples from patients with primary OA (115). A recent study showed that patients with inversion-induced OA had an increased number of PLS3-expressing cells on the medial knee, which is subjected to more mechanical loading, compared to the lateral knee (116). This study hypothesized that the mechanical response of chondrocytes is regulated by the number of PLS3-expressing cells and suggested that Ca^2+^-dependent mechanotransduction between the ECM and actin cytoskeleton *via* PLS3 may play an important role in OA pathogenesis (116). In addition, the RANKL-RANK pathway has also been suggested to be associated with OA (117), and pathological changes such as thickening and sclerosis of subchondral bone and formation of bone redundancy at the joint margins in OA (118) may be associated with increased synthesis of PLS3 (37, 115). The exact role of PLS3 in articular chondrocytes is unclear, and the molecular mechanisms involved in cartilage regulation deserve more attention.

## 5 Perspective on therapy

Considering the obscurity of the role of PLS3 in bone fragility, it remains challenging to accurately envision suitable treatment. Medications that are conventionally designed for the treatment of osteoporosis bone fragility, have shown some success in PLS3 patients although this is based on scarce cases. Bisphosphonates have shown potential in increasing BMD in patients with *PLS3* variants, especially when treated at an early age (7, 44, 80), although a case of atypical femur fracture(AFF) has also been reported (119). Also, in a Dutch cohort of AFFs and early-onset osteoporosis (EOOP), after 9 years of bisphosphonate use, AFF was found in a young male adult with a PLS3 variant, raising concerns about the safety of these agents in the long run in children diagnosed with PLS3 (120). Yet, a 9-year-old Chinese boy diagnosed with OI due to a PLS3 variant, who received bisphosphonate treatment and was followed up for 2 years, showed improved BMD Z-score (121). Improvement was also documented in a 16-year-old boy of Chinese origin with scoliosis and childhood-onset osteoporosis (122). Treatment with teriparatide, a parathyroid hormone analog, increased femoral neck and lumbar spine BMD, which also correlated with increased procollagen type 1 amino-terminal propeptide (P1NP) a marker of bone formation (123). Interestingly, a combination of teriparatide with denosumab, a monoclonal antibody against RANKL, was more effective than teriparatide alone (124). A high dose of vitamin D supplementation has been also found to deliver a therapeutic benefit in increasing BMD (125).

Despite the promising results of the above mentioned therapies on these few patient cases, none of these is yet approved for OI or the specific genetic defect of PLS3. In order to cure the disease, the elimination of the genetic defect will be required by means of gene therapy or stem cell transplantation (126). The adeno-associated virus-mediated delivery has been used to deliver PLS3 in mice with spinal muscle atrophy which extended their viability (35). Currently, gene therapy systems are being developed for delivery specifically to bone (127). Based on future insights in the disease mechanism, it may also become possible to influence relevant or compensatory pathways of PLS3 defects towards homeostatic balance. Chemical inhibition of PLS2 was able to attenuate bone loss in ovariectomized mice and accelerate bone fracture healing (43) whereas the delivery of PLS2 peptides in loaded nanocarriers to osteoclasts was able to inhibit sealing ring formation and resorption (128). This may be a suitable strategy for counteracting the high resorption activity of PLS3 patient osteoclasts. Alternatively, the delivery of compensatory proteins for actin bundling could also in principle relieve actin dysfunction but for this more knowledge about the function of PLS3 in cell-specific cytoskeletal dynamics is required.

## 6 Limitations

A limitation of the current review is the relatively small number of reported patients with *PLS3* variants owing to the mild presentation of the disease which may account for undiagnosed cases. Also, this gene is relatively recently discovered and implemented in diagnostic gene panels. With the current knowledge, we cannot yet concretely explain the presentation of disease symptoms in select tissue types and the variation in their severity in relation to the type of PLS3 defect. Considering the rarity of the disease, it will be beneficial to form large patient cohorts in which the phenotypic variability will be systematically characterized. This may also help to test treatment regimens in order to orient medical professionals with establishing treatment guidelines.

Another limitation is the small number of reported animal models for PLS3. At present, we are still facing major knowledge gaps regarding the function of PLS3 in different cell types and the way their dysfunction ultimately leads to the onset of disease. In order to narrow these gaps, we need to establish gene mouse models which faithfully recapitulate the genetic defects of PLS3 and disease presentation. These will allow the exploration of the PLS3 mechanism in bone homeostasis and other tissue types.

## 7 Conclusion

The involvement of PLS3 in biological processes in bone and its mediation of diseases of the skeletal system is a fascinating new area of research with essential implications for both the field of cytoskeletal biology and skeletal development. Uncovering the complex mechanisms underlying the role and regulation of PLS3 in bone tissue will help us model the processes by which PLS3 regulates the sensing of mechanical load, with the ultimate goal of achieving therapeutic interventions targeting bone development and remodeling. Future studies warrant the exploration of the multifaceted molecular mechanisms by which PLS3 intricately regulates physiological and pathological bone tissue conditions, in order to achieve precise treatments for bone-related diseases.

## Author contributions

WZ, JP and YL contributed to the article structure and performed the writing. LZ designed the drawing of the illustration. GP, EE, NB and DM have contributed to the drafting and critical revision of the paper. All authors contributed to the article and approved the submitted version.
